# Sleep Quality Moderates Age-Related Decline in Hippocampal-Dependent Memory in Midlife Adults

**DOI:** 10.1093/geroni/igaf122.4195

**Published:** 2025-12-31

**Authors:** Kyoung Shin Park, Samantha DuBois, Samuel Kibildis, Hadassah Som-Pimpong, Jarod Vance, Brittany Armstrong, Christopher Wahlheim, Jennifer Etnier

**Affiliations:** Emory University School of Medicine, Atlanta, Georgia, United States; Appalachian State University, Boone, North Carolina, United States; Athens-Clarke County Unified Government, Athens, Georgia, United States; Meharry Medical College, Nashiville, Tennessee, United States; Longwood University, Farmville, Virginia, United States; University of North Carolina at Greensboro, Greensboro, North Carolina, United States; University of North Carolina at Greensboro, Greensboro, North Carolina, United States; University of North Carolina at Greensboro, Greensboro, North Carolina, United States

## Abstract

The hippocampus, critical for learning and memory, is vulnerable to age-related decline. Evidence suggests that sleep is associated with hippocampal integrity and cognitive function in older adults. However, few studies have examined whether sleep quality moderates age-related decline in hippocampal-dependent memory in midlife—a critical time point for detecting preclinical cognitive changes. In this cross-sectional study, we tested the hypothesis that poor sleep quality would be linked to steeper age-related decline in hippocampal-dependent memory performance in midlife. We analyzed data from cognitively normal midlife adults with a family history of Alzheimer’s disease (N = 180; Age= 40–65 years [M = 56.13±6.35 years]; 89% females, MoCA=27.9±1.7) enrolled in a clinical trial (NCT03876314). Participants completed the Pittsburg Sleep Quality Index (PSQI) and Mnemonic Similarity Task (MST)—a sensitive test of hippocampal-dependent memory function. Multiple linear regression was fitted while controlling for potential covariates (e.g., sex, education, APOE-ε4 carriage, and pulse pressure). There was a significant Age × PSQI interaction (b=–0.0017, p=.037) with lure discrimination index (LDI) as the outcome, such that higher PSQI scores (poorer sleep quality) were associated with steeper age-related decline in hippocampal-dependent memory performance. Simple slopes analysis showed that age predicted lower LDI in participants with average (b = −0.01, p=.01) and higher (+1 SD) PSQI (b = −0.01, p<.01), but not those with lower (–1 SD) PSQI (p=.81). These findings suggest the potential role of sleep quality in protecting hippocampal-dependent memory function against age-related decline in midlife and support the utility of the MST for detecting subtle cognitive changes during this critical life stage.

